# XGAP: a uniform and extensible data model and software platform for genotype and phenotype experiments

**DOI:** 10.1186/gb-2010-11-3-r27

**Published:** 2010-03-09

**Authors:** Morris A Swertz, K Joeri van der Velde, Bruno M Tesson, Richard A Scheltema, Danny Arends, Gonzalo Vera, Rudi Alberts, Martijn Dijkstra, Paul Schofield, Klaus Schughart, John M Hancock, Damian Smedley, Katy Wolstencroft, Carole Goble, Engbert O de Brock, Andrew R Jones, Helen E Parkinson, members of the Coordination of Mouse Informatics Resources (CASIMIR), Genotype-To-Phenotype (GEN2PHEN) Consortiums, Ritsert C Jansen

**Affiliations:** 1 Genomics Coordination Center, Department of Genetics, University Medical Center Groningen and University of Groningen, 9700 RB Groningen, The Netherlands; 2 Groningen Bioinformatics Center, University of Groningen, 9750 AA Haren, The Netherlands; 3 EMBL - European Bioinformatics Institute, Hinxton, Wellcome Trust Genome Campus Hinxton, Cambridge CB10 1SD, UK; 4 Experimental Mouse Genetics, Helmholtz Center for Infection Research, Inhoffenstraße 7, D-38124 Braunschweig, Germany; 5 Center for Medical Biomics, University of Groningen, Groningen, A. Deusinglaan 1, 9713 AV Groningen, The Netherlands; 6 Physiological Development and Neuroscience, University of Cambridge, Downing Street, Cambridge CB2 3DY, UK; 7 Bioinformatics Group, MRC Harwell, Harwell, Oxfordshire OX11 0RD, UK; 8 Information Management Group, School of Computer Science, University of Manchester, Kilburn Building, Oxford Road, Manchester M13 9PL, UK; 9 Department of Business and ICT, Faculty of Economics and Business, University of Groningen, 9700 AV Groningen, The Netherlands; 10 Department of Pre-Clinical Veterinary Science and Veterinary Pathology, Faculty of Veterinary Science, University of Liverpool, Liverpool L69 7ZJ, UK

## Abstract

We present an extensible software model for the genotype and phenotype community, XGAP. Readers can download a standard XGAP (http://www.xgap.org) or auto-generate a custom version using MOLGENIS with programming interfaces to R-software and web-services or user interfaces for biologists. XGAP has simple load formats for any type of genotype, epigenotype, transcript, protein, metabolite or other phenotype data. Current functionality includes tools ranging from eQTL analysis in mouse to genome-wide association studies in humans.

## Abstract 2

XGAP, a software platform for the integration and analysis of genotype and phenotype data.

## Background

Modern genetic and genomic technologies provide researchers with unprecedented amounts of raw and processed data. For example, recent genetical genomics [1-3] studies have mapped gene expression (eQTL), protein abundance (pQTL) and metabolite abundance (mQTL) to genetic variation using genome-wide linkage and genome-wide association experiments on various microarray, mass spectrometry and proton nuclear magnetic resonance (NMR) platforms and in a wide range of organisms, including human [4-8], yeast [9,10], mouse [11], rat [12], *Caenorhabditis elegans *[13] and *Arabidopsis thaliana *[14-16].

Understanding these and other high-tech genotype-to-phenotype data is challenging and depends on suitable 'cyber infrastructure' to integrate and analyze data [17,18]: data infrastructures to store and query the data from different organisms, biomolecular profiling technologies, analysis protocols and experimental designs; graphical user interfaces (GUIs) to submit, trace and retrieve these particular data; communicating infrastructure in, for example, R [19], Java and web services to connect to different processing infrastructures for statistical analysis [20-24] and/or integration of background information from public databases [25]; and a simple file format to load and exchange data within and between projects.

Many elements of the required cyber infrastructure are available: The Generic Model Organism Database (GMOD) community developed the Chado schema for sequence, expression and phenotype data [26] and delivered reusable software components like gbrowse [27]; the BioConductor community has produced many analysis packages that include data structures for particular profiling technologies and experimental protocols [28]; and numerous bespoke databases, data models, schemas and formats have been produced, such as the public and private microarray expression databases and exchange formats [29-31]. Some integrated cyber infrastructures are also available: the National Center for Biotechnology Information (NCBI) has launched dbGaP (database of genotypes and phenotypes) [32], a public database to archive genotype and clinical phenotype data from human studies; and the Complex Trait Consortium has launched GeneNetwork [33], a database for mouse genotype, classical phenotype and gene expression phenotype data with tools for 'per-trait' quantitative trait loci (QTL) analysis.

However, a suitable and customizable integration of these elements to support high throughput genotype-to-phenotype experiments is still needed [34]: dbGaP, GeneNetwork and the model organism databases are designed as international repositories and not to serve as general data infrastructure for individual projects; many of the existing bespoke data models are too complicated and specialized, hard to integrate between profiling technologies, or lack software support to easily connect to new analysis tools; and customization of the existing infrastructures dbGaP, GeneNetwork or other international repositories [35,36] or assembly of Bioconductor and generic model organism database components to suit particular experimental designs, organisms and biotechnologies still requires many minor and sometimes major manual changes in the software code that go beyond what individual lab bioinformaticians can or should do, and result in duplicated efforts between labs if attempted.

To fill this gap we here report development of an extensible data infrastructure for genotype and phenotype experiments (XGAP) that is designed as a platform to exchange data and tools and to be easily customized into variants to suit local experimental models. We therefore adopted an alternative software engineering strategy, as outlined in our recent review [37], that enables generation of such software efficiently using three components: a compact and extensible 'standard' model of data and software; a high-level domain-specific language (DSL) to simply describe biology-specific customizations to this software; and a software code generator to automatically translate models and extensions into all low-level program files of the complete working software, building on reusable elements such as listed above as well as general informatics elements and some new/optimized elements that were missing.

Below we detail XGAPs extensible 'standard' software model (XGAP-OM) and evaluate the auto-generated text file exchange format (XGAP-TAB) and customizable database software (XGAP-DB) that should help researchers to quickly use and adapt XGAP as a platform for their genetics and/or *omics experiments (Table 1). Harmonized data representations and programmatic interfaces aim to reduce the need for multiple format convertors and easy sharing of downstream analysis tools via a hub-and-spoke architecture. Use of software auto-generation, implemented using MOLGENIS, aims to ease and speed up customization/variation into new XGAP versions for new biotechnologies and alternative experimental designs while ensuring consistent programming interfaces for the integration and sharing of existing analysis tools. Standardized extension mechanisms should balance between format/interface stability for existing data types and tools, and flexibility to adopt new ones.

**Table 1:** Features of XGAP database for genotype and phenotype experiments

**Store**	Store genotype and phenotype experimental data using only four 'core' data types: *Trait*, *Subject*, *Data*, and *DataElement*. For example: a single-channel microarray reports raw gene expression *Data *for each microarray probe *Trait *and each individual *Subject*. Add information on data provenance by giving details in *Investigation*, *Protocols *and *ProtocolApplications*
	
**Customize**	Customize 'my' XGAP database with extended variants of *Trait *and *Subject*. In the online XGAP demonstrator, *Probe *traits have a sequence and genome location and *Strain *subjects have parent strains and (in)breeding method. Describe extensions using MOLGENIS language and the generator automatically changes XGAP database software to your research
	
**Upload**	Upload data from measurement devices, public databases, collaborating XGAP databases, or a public XGAP repository with community data. Simply download trait information as tab-delimited files from one XGAP and upload it into another; this works because of the uniformity of the core data types (and extensions thereof)
	
**Search**	Search genetical genomics data using the graphical user interface with advanced query tools. The uniformity of the 'code generated' interfaces make it easy to learn and use interfaces for both 'core' data types as well as customized extensions
	
**Analyze**	Analyze data by connecting tools using simple methods in Java, R, Web Services or Internet hyperlinks. For example, map and plot quantitative trait loci in R using XGAP data retrieved via the R interface
	
**Plug-in**	Plug-in the best analysis tools into the user interface so biologists can use them. Bioinformaticians are provided with simple mechanisms to seamlessly add such tools to XGAP, building on the automatically generated GUI and API building blocks
	
**Share**	Share data, customizations, connected analysis tools and user interface plug-ins with the genetical genomics community, using XGAP as exchange platform. For example, the MetaNetwork R package can talk to data in XGAP. This makes it easy for other XGAP owners to also use it

API: application programming interface; GUI: graphical user interface; MOLGENIS: biosoftware generator for MOLecular GENetics Information Systems.

## Minimal and extensible object model

We developed the XGAP object model to uniformly capture the wide variety of (future) genotype and phenotype data, building on generic standard model FuGE (Functional Genomics Experiment) [38] for describing the experimental 'metadata' on samples, protocols and experimental variables of functional genomics experiments, the OBO model (of the Open Biological and Biomedical Ontologies foundry for use of standard and controlled vocabularies and ontologies that ease integration [39], and lessons learned from previous, profiling technology-specific modeling efforts [29].

**Figure 1:**
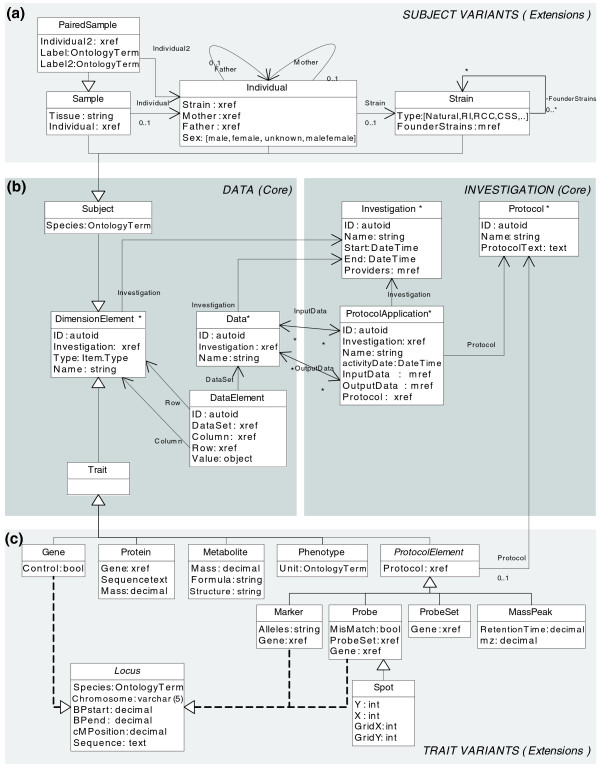
**Extensible genotype and phenotype object model**. Experimental genotype and (molecular) phenotype data can be described using *Subject*, *Trait*, *Data *and *DataElement*; the experimental procedures can be described using *Investigation*, *Protocol *and *ProtocolApplication *(**B)**. Specific attributes and relationships can be added by extending core data types, for example, *Sample *and *Gene *(**A, C**). See Table 2, 3 and 4 for uses of this model. The model is visualized in the Unified Modeling Language (UML): arrows denote relationships (*Data *has a field Investigation that refers to *Investigation *ID); triangle terminated lines denote inheritance (*Metabolite *inherits all properties ID, Name, Type from *Trait*, next to its own attributes Mass, Formula and Structure); triangle terminated dotted lines denote use of interfaces (*Probe *'implements' properties of *Locus*); relationships are shown both as arrows and as properties ('xref' for one-to-many, 'mref' for many-to-many relationships). Asterisks mark FuGE-derived types (for example, Protocol*).

Figure 1b shows the core components of a genotype-to-phenotype investigation: the biological subjects studied (for example, human individuals, mouse strains, plant tissue samples), the biomolecular protocols used (for example, Affymetrix, Illumina, Qiagen, liquid chromatography-mass spectrometry (LC/MS), Orbitrap, NMR), the trait data generated (usually data matrices with, for example, phenotype or transcript abundance data), the additional information on these traits (for example, genome location of a transcript, masses of LC/MS peaks), the wet-lab or computational protocols used (for example, MetaNetwork [22] in the case of QTL and network analysis) and the derived data (for example, QTL likelihood curves).

We describe these biological components using FuGE data types and XGAP extensions thereof. *Investigation *binds all details of an investigation. Each investigation may apply a series of biomolecular [40] and computational [20-23]*Protocols*. The applications of such *Protocols *are termed *ProtocolApplications*, which in the case of computational *Protocols *may require input *Data *and will deliver output *Data*. These *Data *have the form of matrices, the *DataElements *of which have a row and a column index. Each row and column refers to a *DimensionElement*, being a particular *Subject *or a particular *Trait*. Table 2 illustrates the usage of these core data types.

**Table 2:** Use cases of core data types

A growth measurement (*Data*) reports the time (*DataElement*) it took to flower (*Trait*) for an *Arabidopsis *plant (*Subject*)

A two-color microarray result (*Data*) describes raw intensities measured (*DataElement*) for gene transcript probe hybrdization (*Trait*) for each pair of *Arabidopsis *individuals (*Subject*)

A marker measurement (*ProtocolApplication*) resulted in a genetic profile (*Data*) with genotype values (*DataElement*) for each SNP/microsatellite marker (*Trait*) for each human individual (*Subject*)

A genetical genomics stem cell *Investigation *was carried out on 30 recombinant mouse inbred strains (*Subject*). It involved a *ProtocolApplication *of the 'Affymetrix MG-U74Av2' *Protocol *to produce expression profiles (*Data*) for 12,422*16 microarray probes (*Traits*). These profiles consisted of a matrix of signals (*DataElement*) for each Probe (*Traits*) and each InbredStrain (*Subject*). Subsequently, these *Data *were taken as *inputData *in a normalization procedure (*ProtocolApplication*) using RMA normalization *Protocol*, which resulted in *outputData *of normalized profiles (*Data*) of Probe*InbredStrain (Trait*Subject)


RMA: robust multi-array average.

Figure 1a, c shows how the XGAP model can be extended to accommodate details on particular types of subjects and traits in a uniform way. A *Trait *can be a classical phenotype (for example, flowering - the flowering time is stored in the *DataElement*) or a biomolecular phenotype (for example, *Gene *X - its transcript abundance is stored in the *DataElement*). A *Trait *can also be a genotype (for example, *Marker *Y is a genomic feature observation that is stored in the *DataElement*). Genomic traits such as *Gene*, *Marker *and *Probe *all need additional information about their genome *Locus *to be provided. Similarly, a *Subject *can be a single *Sample *(for example, a labeled biomaterial as put on a microarray) and such a sample may originate from one particular *Individual*. It may also be a *PairedSample *when biomaterials come from two individuals - for example, if biomaterial has been pooled as in two-color microarrays. An individual belongs to a particular *Strain*. When new experiments are added new variants of *Trait *and *Subject *can be added in a similar way. Table 3 illustrates the generic usage of these extended data types.

**Table 3:** Use cases of extended data types

*Sample *is a *Subject *with the additional property that 'Tissue' can be specified

*Individual *is a *Subject *with the additional property that relationships with Mother and Father individuals, as well as *Strain*, can be specified

*PairedSample *is a *Sample *with the additional property that 'Dye' has to be specified and which two Subjects (or subclasses such as Individual) are labeled with 'Cy3' and 'Cy5'

An *InbredStrain *is a *Strain *with the additional property that the 'Parents' (mother Individual and father Individual) are specified and the 'type' of inbreeding used

An amplified fragment length polymorphism, microsatellite or SNP *Marker *(is a *Trait*) may refer to genetic and possible genomics location (*Marker *also is a *Locus*)

A correlation computation (*Data*) reports associations (*DataElement*) between *Metabolite *(is a *Trait*); because *Trait *and *Subject *are both extensions of *DimensionElement*, they can be connected to a row and column of *DataElement *interchangeably

Several standard data types were also inherited from FuGE to enable researchers to provide 'Minimum Information' for QTLs and Association Studies such as defined in the MIQAS checklist [41] - a member of the Minimum Information for Biological and Biomedical Investigations (MIBBI) guideline effort [42]. Data types *Action(Application)*, *Software(Application)*, *Equipment(Application) and Parameter(Value) *can be used to describe *Protocol(Application)s *in more detail. For example, a normalization *Protocol *may involve a 'robust multiarray average (RMA) normalization' *Action *that uses Bioconductor 'affy' *Software *[43] with certain *ParameterValues*. Data types *Description*, *BibliographicReferences*, *DatabaseEntry*, *URI*, and *FileAttachment *enable researchers to freely add additional annotations to certain data types - *DimensionElement*, *Investigation*, *Protocol*, *ProtocolApplication*, and *Data*. For example, researchers can annotate a *Gene *with one or more *DatabaseEntries*, referring to unique database accession numbers for automated data integration.

A unique feature of XGAP is the uniform treatment of the various trait and subject annotations. The drawback of allowing users to freely add additional annotations such as described above is that users and tools using metabolite and gene traits, for example, would have to inspect each *Trait *instance to see whether it is actually a metabolite or gene, and how it is annotated. That is why we instead use the object-oriented method of 'inheritance' to explicitly add essential properties to *Trait *and *Subject *variants to make sure that they are described in a uniform way. For example, *Metabolite *extends *Trait*, which explicitly adds properties ID, Name and Type (inherited from *DimensionElement*) to metabolite specific properties Mass, Formula and Structure. See Jones *et al.*[38] for the complete FuGE specifications and Jones and Paton [44] for a discussion on the benefits and drawbacks of alternative mechanisms for supporting extension in object models. Table 4 illustrates the usage of these annotation data types.

**Table 4:** Use cases of annotation data types

A *Gene *in an *Arabidopsis Investigation *can be connected to a *DatabaseEntry *describing a reference to related information in the TAIR database [71] and another *DatabaseEntry *describing a reference to the MIPS database [72]

Each *Individual *in a *C. elegans Investigation *is annotated with an *OntologyTerm *to indicate that it was grown in an environment of either 16°C or 24°C

The *Arabidopsis Investigation *was annotated with the *BibliographicReferences *pointing to the paper describing the investigation and expected results

A *Protocol *describes the 'MapTwoPart' method for QTL mapping and was annotated with the *URI *linking to the 'MetaNetwork R-package', which contains this method, and a *BibliographicReference *pointing to the paper [22,67] that describes the MapTwoPart protocol

A file with a Venn diagram describing the number of masses detected in each population was added as *FileAttachement *to the *Arabidopsis *metabolite *Investigation*

Another feature of XGAP is the uniform treatment of all data on these subjects and traits. To understand basic data in XGAP, newcomers just have to learn that all data are stored as *Data *matrices with each *DataElement *describing an observation on *Subjects *and/or *Traits *(rows × columns). Unlike the proven matrix structures used in MAGE-TAB (tabular format for microarray gene expression experiments) [45], in XGAP these data can be on any *Trait *and/or *Subject *combination, that is, we did not create many variants of *DataElement *to accommodate each combination of *Trait *and *Subject *such as MAGE-TAB's ExpressionDataElement (Probe × Sample), MassSpecDataElement (MassPeak × Sample), eQtlMappingDataElement (Marker × Probe), and so on. Instead, we store all these data using the generic type *DataElement *and limit extension to *Trait *and *Subject *only. This avoids the (combinatorial) explosion of *DataElement *extensions so researchers can provide basic data as common data matrices (of *DataElements*) and can still add particular annotations flexibly to the matrix row and columns to allow for (new) biotechnologies as demonstrated in the various *Trait *extensions in Figure 1. Keeping this simple and uniform data structure greatly enhances data and software (re)usability and hence productivity, in line with the findings by Brazma *et al. *[29] and Rayner *et al. *[45] that the simple tabular structures underlying biological data should be exploited instead of making it overly complicated.

After structural homogenization, such as provided by FuGE and XGAP, semantic queries are the remaining major barrier for integration of experimental metadata. This requires ontologies that describe the properties of the materials and also descriptions of experimental processes, data and instruments. The former are provided by species-specific ontologies that are available from various sources. The Ontology for BioMedical investigation [46] may provide a solution for the experimental descriptors and is being used in this context by, for example, the Immune Epitope Database [47]. To enable researchers to use these well understood descriptors, XGAP inherits from FuGE the mechanism of 'annotations', a special field to link any data object to one or more ontology terms. For example, researchers can annotate a *Gene *with one or more *OntologyTerms *if required, referring to standard ontology terms from OBO [39] or ontology terms defined locally.

## Simple text-file format for data exchange

To enable data exchange using the XGAP model, we produced a simple text-file format (XGAP-TAB) based on the experience that for data formats to be used, data files should be easily created using simple Excel and text editor tools and closely resemble existing practices. This format is automatically derived from the model by requiring that all annotations on *Investigations*, *Protocols*, *Traits*, *Subjects*, and extensions thereof, are described as delimited text files (one file per data type) with columns matching the properties described in the object model and each row describing one data instance. Optionally, sets of *DataElements *can also be formatted as separate text matrices with row and column names matching these in the *Trait *and *Subject *annotation files, and with each matrix value matching one *DataElement*. The dimensions of each data matrix are then listed by a row in the annotations on *Data*.

**Figure 2:**
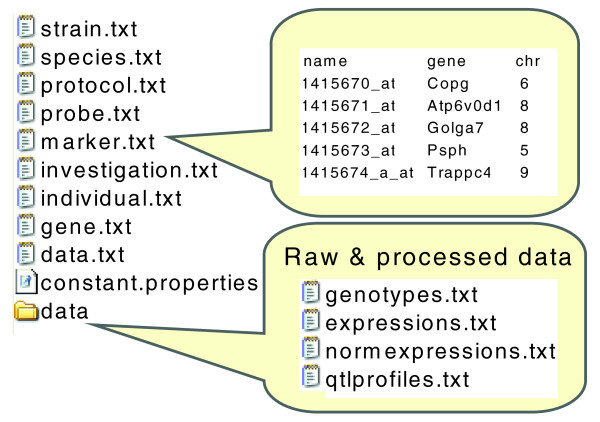
**Simple text file format**. A whole investigation can be stored by using easy-to-create tabular text files for annotations or matrix-shaped text files for raw and processed data. Each 'annotation' file relates to one data type in the object model shown in Figure 1 - for example, the rows in the file 'probe.txt' will have the columns named in data type 'Probe'. Each 'data' file contains data elements and has row names and column names referring to annotation files - for example, 'genotypes.txt' may refer to 'marker.txt' names as row names and 'individual.txt' names as column names. If convenient, constant values can be described in the constant.properties file such as 'species_name'.

Figure 2 shows one investigation in the XGAP tabular data format with one delimited text file per data type - that is, there are files named 'probe.txt' and 'individual.txt', with each row describing a microarray probe or individual, respectively - and one text matrix file per set of *DataElements *- that is, there are files named 'data/expressions.txt' and 'data/genotypes.txt'. The properties of each data matrix is then described in 'data.txt'; that is, for the 'data/expressions.txt' there is a row in 'data.txt' that says that its columns refer to 'individual.txt', that its rows refer to 'probe.txt' and that its values are 'decimal'. Raw data sets and data sets in other formats can be retained in a directory labeled 'original'.

After proving its value in several proprietary projects, a growing array of public data sets are now available at [48] demonstrating the use of XGAP-TAB [8,11,13,14,49,50].

## Easy to customize software infrastructure

A pilot software infrastructure is available at [51] to help genotype-to-phenotype researchers to adopt XGAP as a backbone for their data and tool integration. We chose to use the MOLGENIS toolkit (biosoftware generator for MOLecular GENetics Information Systems; see Materials and methods) to auto-generate from the XGAP model: 1, an SQL (Structured Query Language for relational databases) file with all necessary statements for setting up your own, customized variant of the XGAP database; 2, application programming interfaces (APIs) in R, Java and Web Services that allow bioinformaticians to plug-in their R processing scripts, Taverna workflows [25,52,53] and other tools; 3, a bespoke web-based graphical user interface (GUI) by which researchers can submit and retrieve data and run plugged-in tools; and 4, import/export wizards to (un)load and validate data sets exchanged in XGAP-TAB format. The auto-generation process can be repeated to quickly customize XGAP from an extended model, for example, to accommodate a particular new type of measurement technology or experimental design.

### Graphical user interface

**Figure 3:**
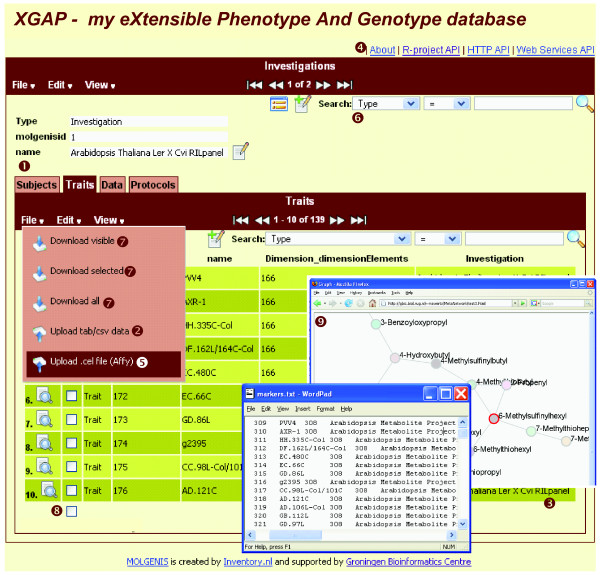
**Graphical User Interfaces**. A user interface enables biologists to add and retrieve data and run integrated tools. Genotype and phenotype information can be explored by investigation, subjects, traits or data. Hyperlinks following cross-references of the object model point to related information. Items indicated by 1-9 are described in the main text. See Table 5 for uses of this GUI. See also our online demonstrator at [51].

Figure 3 shows the GUI to upload, manage, find and download genotype and phenotype data to the database. The GUI is generated with a uniform 'look-and-feel', thereby lowering the barrier for novice users. Investigations can be described with all subjects, traits, data and protocol applications involved (1). (The numbers refer to steps in the figure.) Data can be entered using either the edit boxes or using menu-option 'file|upload' (2). This option enables upload of whole lists of traits and subjects from a simple tab-delimited format (3), which can easily be produced with Excel or R; MOLGENIS automatically generates online documentation describing the expected format (4). Subsequently, the protocol applications involved can be added with the resulting raw data (for example, genetic fingerprints, expression profiles) and processed data (for example, normalized profiles, QTL profiles, metabolic networks). These data can be uploaded, again using the common tab-delimited format or custom parsers (5) that bioinformaticians can 'plug-in' for specific file formats (for example, Affymetrix CEL files). The software behind the GUI checks the relationships between subjects, traits, and data elements so no 'orphaned' data are loaded into the database - for example, genetic fingerprint data cannot be added before all information is uploaded on the markers and subjects involved. Standard paths through the data upload process are employed to ensure that only complete and valid data are uploaded and to provide a consistent user experience.

Biologists can use the graphical user interface to navigate and retrieve available data for analysis. They can use the advanced search options (6) to find certain traits, subjects, or data. Using menu option 'file|download' (7) they can download visible/selected (8) data as tab-delimited files to analyze them in third party software. Bioinformaticians can 'plug-in' a custom-built screen (see 'customization' section) that allows processing of selected data inside the GUI, for example, visualizing a correlation matrix as a graph (9) without the additional steps of downloading data and uploading it into another tool. Biologists can create link-outs to related information, for example, to probes in GeneNetwork.org (not shown). Table 5 summarizes use cases of the graphical user interface.

**Table 5:** Use cases of the graphical user interface for biologists

Navigate all *Investigations*, and for each *Investigation*, see the *Assays *and available *Data*

Select a *Gene *and find all *Investigations *in which this *Gene *is regulated as suggested by significant eQTL *Data *(*P*-value < 0.001)

For a given *Locus*, select all *Genes *that have QTL *Data *mapping 'in *trans*'; and this may be regulated by this *Locus*, for example, absolute(QTL locus - gene locus) > 10 Mb and QTL *P*-value < 0.001

Download a selection of raw gene expression *Data *as a tab-delimited file (to import into other software)

Upload *Investigation *information from tab-delimited files

Upload Affymetrix *Assays *using custom *.CEL/*.CDF file readers

Plot highly correlated metabolic network *Data *in a network visualization graph

Define security levels for *Assays*/*Investigations *to ensure that appropriate data can be viewed only by collaborators, and not by other people

A *MassPeak *has been identified to be 'proline' and we can follow the link-out *URI *to Pubchem [46], because it was annotated to have 'cid' 614, to find information on structure, activity, toxicology, and more

### Application programming interfaces

*De facto *standard analysis tools are emerging, for example, tools for transcript data [20,21,24] or metabolite abundance data [22] to mention just a few. These tools are typically implemented using the open source software for statistical analysis and graphics named R [19]. Bioinformaticians can connect their particular R or Java programs to the XGAP database using an API with similar functionality to the GUI, that is, using simple commands like 'find', 'add' and 'update' (R/API, Java/API). Scripts in other programming languages and workflow tools like Taverna [53] can use web services (SOAP/API) or a simple hyperlink-based interface (HTTP/API), for example, http://my-xgap/api/find/Data?investigation=1 returns all data in investigation '1'. On top of this, conversion tools have been added to the R interface to read and write XGAP data to the widely used R/qtl package [24].

**Figure 4:**
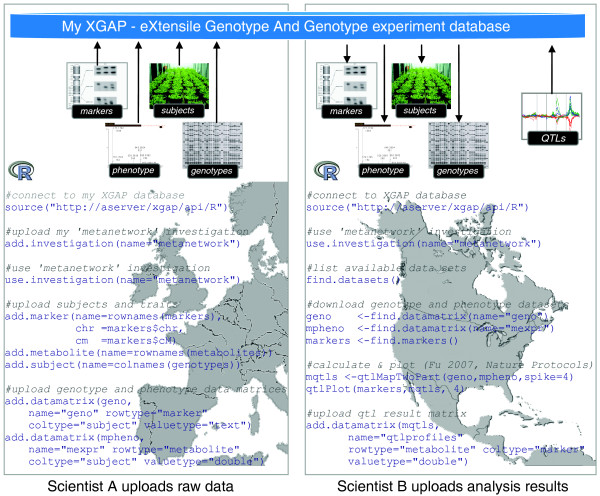
**Application programming interfaces**. APIs enable bioinformaticians to integrate data and tools with XGAP using web services, R-project language, Java, or simple HTTP hyperlinks. The figure shows how scientists can use the R/API to upload raw investigation data (Scientist A) so another researcher can download these data and immediately use it for the calculation of QTL profiles and upload the results thereof back to the XGAP database for use by another collaborator (Scientist B). Note how 'add.datamatrix' enables flexible upload of matrices for any *Subject *or *Trait *combination; this function adds one row to *Data *for each matrix, and as many rows to *DataElement *as the matrix has cells. See Table 6 for uses of these APIs.

Figure 4 demonstrates how researchers can use the R/API to download (or upload) all trait/subject/data involved in their investigation from (or to) their XGAP database for (after) analysis in R. When XGAP is customized with additional data type variants, the APIs are automatically extended in the XGAP database instances by re-running the MOLGENIS generator, thus also allowing interaction with new data types in a uniform way. These new types can then be used as standard parameters for new analysis software written in R and Java. Table 6 summarizes use of the application programming interface.

**Table 6:** Use cases of the application programming interface for bioinformaticians

In R, parse a set of tab-delimited *Marker*, *Genotype *and *Trait *files and load them into the database (R/API)

In R, retrieve all *Traits*, *Markers*, expression *Data*, and genotype *Data *from an investigation as data matrices, before QTL mapping with MetaNetwork (R/API)

In Java, retrieve a list of QTL profile correlation *Data *to show them as a regulatory network graph (J/API)

In Java, customize generated file readers to load specific file formats (J/API)

In Taverna, retrieve *Genes *from XGAP to find pathway information in KEGG (WS/API)

In Python, retrieve a list of QTL mapping *Data *using a hyperlink to XGAP (HTTP/API)


KEGG: Kyoto Encyclopedia of Genes and Genomes.

### Import/export wizards

A generated import tool takes care of checking the consistency of all traits, subjects and data that are provided in XGAP-TAB text files and loads them into the database. The entries in all files should be correctly linked, the data must be imported in the right order and the names and IDs need to be resolved between all the annotation files to check and link genes, microarray probes and gene expression to the data. The import program takes care of all these issues (conversion, relationship checks, dependency ordering, and so on). Moreover, the import program supports 'transactions', which ensures that all data inserts are rolled back if an import fails halfway, preventing incomplete or incorrect investigation data to be stored in the database. In a similar way, an export wizard is provided to download investigation data as a zipped directory of XGAP-TAB files.

When XGAP is customized with additional data type variants, the import/export program is automatically extended by the MOLGENIS generator, 'future-proofing' the data format for new biotechnological profiling platforms. Moreover, the auto-generated import program can also be used as a template for parsers of proprietary data formats, such as implemented in parsers for the PED/MAP, HapMap, and GeneNetwork data. Collaborations are underway within EBI and GEN2PHEN to also enable import/export of MAGE-TAB [45] files, the standard format for microarray experiments, of PAGE-OM [54] files, a specialized format for genome-variation oriented genotype-to-phenotype experiments, and of ISA-TAB [55] files, a generalized evolution of MAGE-TAB to represent all experimental metadata on any investigation, study and assay designed to be FuGE compatible. Also, convertors to ease retrieval and submission to public repositories like dbGaP are under development. It is envisaged that integration of all these formats will enable integrated analysis of experimental data from, for example, mouse and human experiments using various biotechnology platforms, which was previously near impossible for biological labs to implement.

### Customizing XGAP

**Figure 5:**
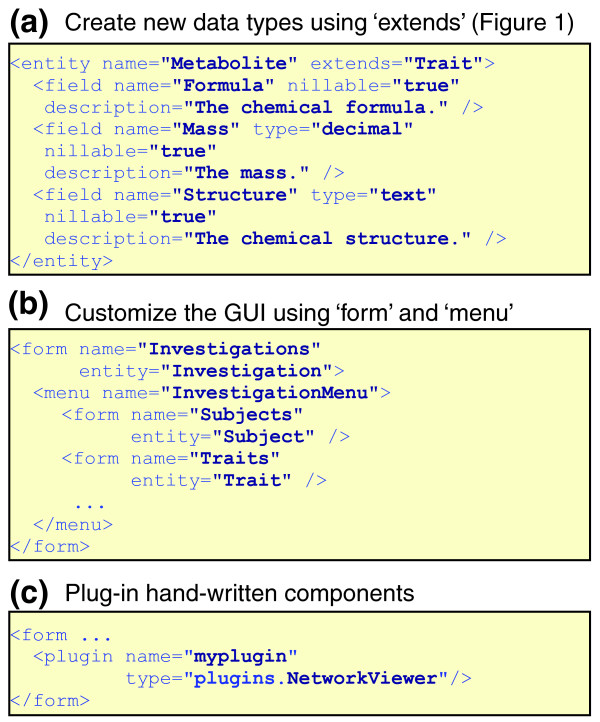
**Customizing XGAP**. A file in MOLGENIS domain-specific language is used to describe and customize the XGAP database infrastructure in a few lines. **(a) **Shows how the addition of a *Metabolite *data entity as a new variant of *Trait *takes only a few lines in this DSL. **(b) **Shows how the GUI can be customized to suit a particular experimental process. **(c) **Shows how programmers can add a 'plug-in' program that is not generated by MOLGENIS but written by hand in Java.

Customizations and extensions of the XGAP object model can be described in a single text file using MOLGENIS [37,56] DSL. On the push of a button, the MOLGENIS generator instantly produces an extended version of the XGAP database software from this DSL file. A regression test procedure assists XGAP developers to ensure their extensions do not break the XGAP exchange format. Figure 5a shows how the addition of a *Metabolite *data entity as a new variant of *Trait *takes only a few lines in this DSL. Figure 5b shows how the GUI can be customized to suit a particular experimental process. Figure 5c shows how programmers can add a 'plug-in' program that is not generated by MOLGENIS but written by hand in Java (for example, a viewer that plots QTL profiles interactively). Moreover, use of Cascading Style Sheets (CSS) enables research projects to completely customize the look and feel of their XGAP.

All XGAP and MOLGENIS software can be downloaded for free under the terms of the open source license LGPL. Extended documentation on XGAP and MOLGENIS customization is available online at the XGAP and MOLGENIS wikis [51,57].

## Conclusions

In this paper we report a minimal and extensible data infrastructure for the management and exchange of genotype-to-phenotype experiments, including an object model for genotype and phenotype data (XGAP-OM), a simple file format to exchange data using this model (XGAP-TAB) and easy-to-customize database software (XGAP-DB) that will help groups to directly use and adapt XGAP as a platform for their particular experimental data and analysis protocols.

We successfully evaluated the XGAP model and software in a broad range of experiments: array data (gene expression, including tiling arrays for detection of alternative splicing, ChIP-on-chip for methylation, andgenotyping arrays for SNP detection); proteomics and metabolomics data (liquid chromatography time of flight mass spectrometry (LC-QTOF MS), NMR); classical phenotype assays [8,11,13,15,49,50,58,59]; other assays for detection of genetic markers; and annotation information for panel, gene, sample and clone. Non-technical partners successfully evaluated the practical utility by independently formatting and loading parts of their consortium data: EU-CASIMIR (for mouse; Table 7), EU-GEN2PHEN (for human; Table 7), EU-PANACEA (for *C. elegans*) and IOP-Brassica (for plants). A public subset of these data sets is available for download at [51]. When needed we could quickly add customizations to the model, building on the general schema, and then use MOLGENIS to generate a new version of the software at the push of a button, for example, to support *NMR *methods as an extended type of *Trait *[60]. Furthermore we successfully integrated processing tools, such as a two-way communication with R/QTL [24] enabling QTL mapping on XGAP stored genotypes and phenotypes with QTL results stored back into XGAP.

**Table 7:** XGAP participating consortia

Consortium	Remit
CASIMIR	The collection and distribution of large volumes of complex data typical of functional genomics is carried out by an increasing number of disseminated databases of hugely variable scale and scope. Combined analysis of highly distributed datasets provides much of the power of the approach of functional genomics, but depends on databases' ability to exchange data with each other and on analytical tools with semantic and structural integrity. Agreement on the standards adopted by databases will inevitably be a matter of community consensus and to that end a recent coordination action funded by the European Commission, CASIMIR [70], is engaged in a community consultation on the nature of the technical and semantic standards needed. What has already become clear in use-case studies conducted so far is that whatever standards are adopted, they will inevitably remain dynamic and continue to develop, particularly as new data types are collected. Crucially, they should allow the open-ended development of analytical and data-mining software, while integration of efforts to agree such standards and develop new software is essential
	
GEN2PHEN	Currently available genotype-to-phenotype (G2P) databases are few and far between, have great diversity of design, and limited or no interoperability between them. This arrangement provides no convenient way to populate the databases, no easy way to exchange, compare or integrate their content, and absolutely no way to search the totality of gathered information. In this context, the European Commission has recently funded the GEN2PHEN project [55], which intends to significantly improve the database infrastructure available within Europe for the collation, storage, and analysis of human and model-organism G2P data. This will be achieved by first developing various cutting-edge solutions, and then deploying these in conjunction with proven concepts, so as to transform the current elementary G2P database reality into a powerful networked hierarchy of interlinked databases, tools and standards

Based on these experiences, we expect use of XGAP to help the community of genome-to-phenome researchers to share data and tools, notwithstanding large variations in their research aims. The XGAP data format can be used to represent and exchange all raw, intermediate and result data associated with an investigation, and an XGAP database, for instance, can be used as a platform to share both data and computational protocols (for example, written in the R statistical language) associated with a research publication in an open format. We envision a directory service to which XGAP users can publish metadata on their investigations either manually or automatically by configuring this option in the XGAP administration user interface. This directory service can then be used as an entry point for federated querying between the community of XGAPs to share data and tools.

Groups that already have an infrastructure can assimilate XGAP to ease evolution of their existing software. Next to their existing user tools, they can 'rewire' algorithms and visual tools to also use the MOLGENIS APIs as data backend. Thus, researchers still have the same features as before, plus the features provided by the generated infrastructure (for example, data management GUIs, R/API) and connected tools (for example, R packages developed elsewhere). Moreover, much less software code needs to be maintained by hand when replacing hand-written parts by MOLGENIS-generated parts, allowing software engineers to add new features for researchers much more rapidly.

We invite the broader community to join our efforts at the public XGAP.org wiki, mailing list and source code versioning system to evolve and share the best XGAP customizations and GUI/API 'plug-in' enhancements, to support the growing range of profiling technologies, create data pipelines between repositories, and to push developments in the directions that will most benefit research.

## Materials and methods

Software modeling, auto-generation/configuration and component toolboxes are increasingly used in bioinformatics to speed up (bespoke) biological software development; see our recent review [37]. For XGAP we required a software toolbox providing query interfaces, data management interfaces, programming interfaces to R and web services, simple data exchange formats and a minimal requirement of programming knowledge. The MOLGENIS modeling language and software generator toolbox [37,56] was chosen as it combines all these features.

Several alternative toolboxes were evaluated: BioMart [57,61] and InterMine [62] generate powerful query interfaces for existing data but are not suited for data management; Omixed [63] generates programmatic interfaces onto databases, including a security layer, but lacks user interfaces; PEDRO/Pierre [64] generates data entry and retrieval user interfaces but lacks programmatic interfaces; and general generators such as AndroMDA [65] and Ruby-on-Rails [66] require much more programming/configuration efforts compared to tools specific to the biological domain. Turnkey [67] seemed to be closest to our needs: it emerged from the GMOD community having GUI and SOAP interfaces but lacks auto-generation of R interfaces and a file exchange format.

**Figure 6:**
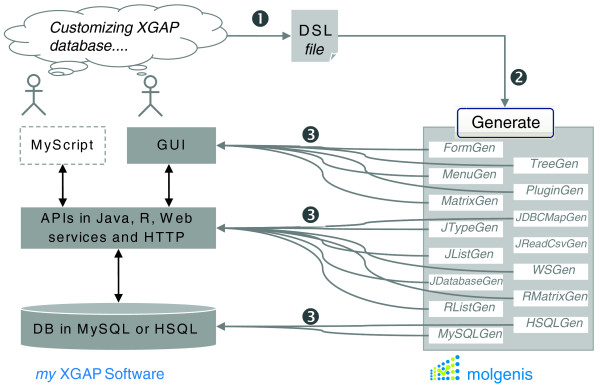
**Auto-generation of XGAP software**. Open source generator tools are used to produce a customized XGAP software infrastructure. 1, The XGAP object model is described using the MOLGENIS' little modeling language (Figure 4). 2, Central software termed MolgenisGenerate runs several generators, building on the MOLGENIS catalogue of reusable assets. 3, At the push of the button, the software code for a working XGAP implementation is automatically generated from the DSL file. GUI and APIs provide simple tools to add and retrieve data, while the reusable assets of MOLGENIS hide the complexity normally needed to implement such tools. For customization, only simple changes to the XGAP model file are required; the MOLGENIS generator takes care of rewriting all the necessary files of SQL and Java software code, saving time and ensuring a consistent quality.

Figure 6 summarizes how MOLGENIS generates the XGAP database software in three layers: database, API and GUI. MOLGENIS either generates a high-performance 'server' edition, which requires installation on server software, or a limited 'standalone' edition that runs on a desktop computer without any additional configuration. The database layer is generated as SQL files with 'database CREATE statements' that are loaded into either MySQL (server), PostgreSQL (server) or HSQLDB (standalone). Each data type in the XGAP object model (Figure 1) is mapped to its own table - for example, there is a 'Trait' table. Each inheritance adds another table, for example, each *Gene *has an entry in the 'Gene' table and also in the 'Trait' table. One-to-many cross-references between data types are mapped as foreign keys - for example, *Data *has a numeric field called 'Investigation' that must refer to the *foreign key *'molgenisid' of *Investigation*. Many-to-many cross-references are mapped via a 'link-table' - for example, an additional table '*mref_import_data' *is generated for two foreign keys to *Data *and *ProtocolApplication*, respectively, to model the *importData *relationship between them. The API layer is generated as Java files either served via Tomcat (server) or Jetty (standalone). A Java class is generated for each data type - for example, there is a class *Gene*. All data can be queried programmatically via a central *Database *class, that is, command *db.find(Gene.class) *returns all *Gene *objects in the database. To enhance performance, the API uses the 'batched' update methods of Java's DataBase Connectivity (JDBC) package and the 'multi-row-syntax' of MySQL to allow inserts of 10,000s of data entries in a single command, an optimization that is 5 to 15 times quicker than standard one-by-one updates. The Java/API is exposed with a SOAP/API, HTTP/API and R/API, so XGAP can also be accessed via web service tools like Taverna, HTTP or R, respectively (accessible via hyperlinks in the GUI). The GUI layer is also generated as Java files. The GUI includes classes for each Menu and Form - for example, the *InvestigationForm *class generates a view- and editform for investigations in the GUI. The generation is steered from one XML file written in MOLGENIS DSL (partially shown in Figure 5). To enable FuGE extension, the FuGE model was automatically translated into MOLGENIS DSL. We therefore first downloaded the FuGE v1 MagicDraw file from [68], exported from MagicDraw to XMI 2.1, parsed the XMI using the EMF parser from Eclipse [69] and then automatically translated it into MOLGENIS DSL using a newly built XmiToMolgenis tool. Compatibility with the FuGE standard is ensured via inheritance; that is, *Investigation*, *Protocol*, *ProtocolApplication*, *Data *and *DimensionElement *in XGAP all extend FuGE data types of the same name. Further implementation details can be found at [51,57].

## Abbreviations

API: application programming interface; dbGaP: database of genotypes and phenotypes; DSL: domain-specific computer language; FuGE: Functional Genomics Experiment model; GMOD: Generic Model Organism Database; GUI: graphical user interface; LC/MS: liquid chromatography-mass spectrometry; MAGE-TAB: tabular format for microarray gene expression experiments; MOLGENIS: biosoftware generator for MOLecular GENetics Information Systems; NMR: proton nuclear magnetic resonance; QTL: quantitative trait locus; SOAP: web services using simple object access protocol; SQL: Structured Query Language for relational databases; XGAP: eXtensible Genotype And Phenotype platform.

## Authors' contributions

MAS, ARJ, PS, KS, JMH, DS, EOB, HEP and RCJ compiled the functional requirements for the XGAP community platform and drafted the extensible data model. MAS, KJV, BMT, RAS, and MD refined and implemented the model using MOLGENIS, and added all parsers, and user interfaces. MAS and KW implemented Taverna compatible web services and GV, DA, KJV and MS implemented R-services. MAS, HEP and RCJ drafted the manuscript. All authors evaluated XGAP components in various settings. All authors read and approved the final manuscript.
